# Brain Somatic Variant in Ras-Like Small GTPase *RALA* Causes Focal Cortical Dysplasia Type II

**DOI:** 10.3389/fnbeh.2022.919485

**Published:** 2022-06-30

**Authors:** Han Xu, Kai Gao, Qingzhu Liu, Tianshuang Wang, Zhongbin Zhang, Lixin Cai, Ye Wu, Yuwu Jiang

**Affiliations:** ^1^Department of Pediatrics, Peking University First Hospital, Beijing, China; ^2^Children Epilepsy Center, Peking University First Hospital, Beijing, China; ^3^Beijing Key Laboratory of Molecular Diagnosis and Study on Pediatric Genetic Diseases, Beijing, China; ^4^Key Laboratory for Neuroscience, Ministry of Education/National Health and Family Planning Commission, Peking University, Beijing, China; ^5^Institute for Brain Disorders, Beijing, China

**Keywords:** pediatric drug-resistant epilepsy, FCD II, somatic mutation, RalA, mTOR pathway

## Abstract

**Purpose:**

In our group’s previous study, we performed deep whole-exome sequencing and targeted amplicon sequencing in the postoperative brain tissue of epilepsy patients with focal cortical dysplasia type II (FCD II). We identified the first somatic variant of *RALA* in the brain tissue of a child with FCD type IIb. *RALA* encodes a small GTPase of the Ras superfamily. To date, the role of *RALA* in brain development is not yet known. In this study, we reported that the *RALA* somatic variant led to FCD type II through activation of the mammalian target of rapamycin (mTOR) pathways.

**Materials and Methods:**

HEK293T cells were transfected *in vitro* to analyze the expression of the RalA protein, as well as phosphorylated S6 (P-S6), one of the major markers of mTOR pathway activation, RalA GTPase activity, and the interaction between RalA and its downstream binding effectors. *In vivo*, wild-type, and mutant *RALA* plasmids were transfected into the local cortex of mice using *in utero* electroporation to evaluate the effect of *RALA* c.G482A on neuronal migration.

**Results:**

The *RALA* c.G482A mutation increased RalA protein expression, the abnormal activation of the mTOR pathways, RalA GTPase activity, and binding to downstream effectors. *RALA* c.G482A local transfection in the embryonic brain *in utero* induced abnormal cortical neuron migration in mice.

**Conclusion:**

This study demonstrated for the first time that the somatic gain-of-function variant of *RALA* activates the mTOR pathway and leads to neuronal migration disorders in the brain, facilitating the development of FCD II. Therefore, *RALA* brain somatic mutation may be one of the pathogenic mechanisms leading to FCD II, which is always related to drug-resistant epilepsy in children. However, more somatic variations of this gene are required to be confirmed in more FCD II patient brain samples.

## Introduction

Malformation of cortical development (MCD) is a common cause of drug-refractory epilepsy in children. Focal cortical dysplasia (FCD), a type of MCD, is characterized by the impaired migration and proliferation of localized cortical neurons during embryonic development, and this is accompanied by abnormal cell proliferation (Mariasavina et al., 2020). In 2011, the International League Against Epilepsy (ILAE) proposed a classification consensus of FCD that divided FCD into three categories (Ingmar et al., 2011), among which FCD II has received widespread attention due to its unique pathological features. FCD II is associated with specific cytological abnormalities, such as dysmorphic neurons (DNs) and balloon cells (BCs), in addition to disrupted cortical lamination. FCD II was further divided into two subtypes, FCD IIa with dysmorphic neurons but without balloon cells and FCD IIb with both dysmorphic neurons and balloon cells. Evidence from research statistics shows that FCD accounts for approximately 75% of the surgically resected MCD brain tissue samples from children undergoing epilepsy surgery, with FCD II being the most common pathological type, accounting for approximately 45.3% (Blumcke et al., 2017).

The underlying molecular genetic etiology of FCD II remains unclarified, but recent genetic studies have shown that somatic mutations in the mammalian target of rapamycin (mTOR) pathway gene have a pivotal role in the pathogenesis of FCD II. Through deep sequencing of surgically resected FCD II brain tissue samples and using matched peripheral blood cell sequencing results of patients as a control, somatic variants were detected in 10–63% of the brain tissues, with mutated genes including *MTOR*, *PIK3CA*, *AKT3*, *RHEB*, *DEPDC5*, *NPRL2*, *NPRL3*, *TSC2*, and *PTEN* primarily located in the mTOR pathways (Lim et al., 2015; Nakashima et al., 2015; Moller et al., 2016; Baulac et al., 2019; Oegema et al., 2020; Blumcke et al., 2021). In addition, the variant allele frequency (VAF) in the tissues was only 0.93–12.63% (Marsan and Baulac, 2018; Baulac et al., 2019; Zhang et al., 2020). Among them, somatic activating mutations in the *MTOR* gene accounted for approximately 15.6–46% of the FCD samples, which is the most common somatic genetic cause of FCD II at present (Lim et al., 2015). mTOR is an evolutionarily conserved serine/threonine-protein kinase, and the PI3K-AKT-mTOR signaling pathway is one of the vital intracellular signaling pathways that regulate cellular transcription, translation, metabolism, proliferation, and migration. When external stimuli act on cells, PI3K can be phosphorylated, and this in turn phosphorylates AKT. Activated AKT inhibits the TSC1/TSC2 complex, and this deactivates the downstream Rheb, leading to the activation of the mTOR complex 1 (mTORC1). Then, the downstream substrates, namely, P70-S6K and S6, are phosphorylated, exerting regulatory effects on protein synthesis, proliferation, and autophagy. In the central nervous system (CNS), the mTOR pathway is involved in regulating neural development, neuronal morphology, neural circuits, and synaptic plasticity (Saxton and Sabatini, 2017). Nevertheless, pathogenic mutations have not been detected in more than half of FCD II brain tissues, and it is unclear whether the occurrence of FCD II is associated with somatic mutations in other pathway-related genes or new mTOR pathway-regulated genes.

The somatic variant of the Ras-Like Proto-Oncogene A gene (*RALA*, c.G482A, p.Arg161Gln) with a VAF of 5.50% was detected for the first time in surgically resected FCD IIb brain tissue by our group earlier, and the activation of the mTOR pathway was also confirmed in the lesions (Zhang et al., 2020). *RALA* encodes a small GTPase of the Ras superfamily, RalA, that is implicated in a range of biological functions including metabolic and transcriptional regulation. In the CNS, RalA is involved in neuronal development, plasticity, polarization, migration, and renewal of synaptic vesicles as well as NMDA, AMPA, and dopamine receptor regulation (Teodoro et al., 2013; Zheng et al., 2016). Previous studies have focused on the role of *RALA* variants in tumor cell proliferation and metastasis, but *RALA* has never been reported to be associated with FCD. In this study, we performed functional studies on the newly discovered somatic variant of *RALA* to explore the effect of this variant on the PI3K-AKT-mTOR signaling pathway and thus on neuronal proliferation and migration. Using in-depth studies of the *RALA* variant, we expected to provide significant clues for further interpretation of the pathogenesis of FCD and the mechanism of epileptogenesis.

## Materials and Methods

### Clinical Data Collection

This study was approved by the Ethics Committee of Peking University First Hospital, and informed consent was signed by the guardians of the participant. Clinical data of the child were collected, including gender, age, epilepsy seizure symptoms, video-electroencephalogram (V-EEG), MRI imaging, and postoperative histopathology.

### Plasmid Constructs for Generating the Wild-Type and the Mutant *RALA*

GV141 Flag-tagged wild-type *RALA* (NM_005402) and GV141 Flag-tagged mutant *RALA* constructs were purchased from the GeneChem Company in Shanghai. After generating the mutant constructs, we checked the full sequence of the coding region for each construct and found no secondary missense or truncated mutation.

### Cell Culture, Transfection, and Western Blotting

Human embryonic kidney 293T (HEK293T) cells were cultured in Dulbecco’s Modified Eagle’s Medium (DMEM) supplemented with 100 units/ml of penicillin, 100 mg/ml of streptomycin, and 10% of fetal bovine serum at 37°C and 5% CO_2_. The cells were transfected with flag-tagged wild-type and mutant *RALA* plasmids while growing to a 70% density. For the immunoblotting, the cells were lysed to extract the total proteins. The membranes were incubated with primary antibodies including anti-phospho-S6 ribosomal protein, anti-S6 ribosomal protein, anti-β-actin, and anti-RalA in TBST overnight at 4°C after electrophoresis. Then, the secondary antibody was incubated and developed for imaging.

### Quantitative Real-Time PCR Analysis

The total RNA was isolated and transcribed into cDNA. Quantitative real-time PCR (qPCR) was performed using the qPCR Kit (Promega) with specific primers designed for the target gene and housekeeping gene. GAPDH was used as the internal control.

### Protein Purification and the RalA GTPase Assays

Flag-tagged proteins were purified using Pierce DYKDDDDK magnetic agarose (Thermo Fisher Scientific) according to the manufacturer’s protocol. Protein purity was assessed using standard SDS-PAGE blotting. The protein concentration was quantified and normalized among samples in an elution buffer prior to use in the assays. The GTPase activity of 5 μg of purified, recombinant proteins was assessed using the GTPase-Glo Assay. The luminescence was quantified using a microplate reader.

### RalA Effector Binding Assays

The binding of the purified, recombinant proteins to a proprietary Ral effector protein was assessed using the RalA G-LISA Activation Assay Kit following the protocols. In brief, purified RalA protein was incubated in the presence or absence of 15 μM of GTP for 1.5 h at 25°C, and then 50 ng of the purified RalA/GTP mixture was applied to the Ral-BP binding plate.

### Immunofluorescence of the Focal Cortical Dysplasia Brain Tissues

The FCD brain specimens were collected after surgery. Surgical tissues were fixed in 4% paraformaldehyde (PFA) overnight, cryoprotected overnight in 20% buffered sucrose, and made into OCT-embedded tissue blocks. Then, cryostat-cut sections (20 μm thick) were performed. Then, cryostat-cut sections were immune-stained with anti-NeuN/SMI-311/Vimentin (V9) antibodies, the mouse antibody to RalA, and then stained with the Alexa Fluor 488-conjugated and Alexa Fluor 568-conjugated secondary antibodies separately. Then, the images were scanned and analyzed using a laser scanning confocal microscope.

### Tandem Mass Tag-Labeled Quantitative Proteome Assay

The transfected cell samples were prepared according to the requirements described in the literature (Wiśniewski et al., 2009; Carolyn et al., 2015; Gillette et al., 2020), and three biological replicates were set for each sample group. Quantitative proteomics analysis was performed by Novogene Company.

### *In utero* Electroporation and Image Analysis

Pregnant ICR mice at embryonic day 14.5 (E14.5) were used in this experiment, and the detailed procedures are described in the previous literature (Wang and Mei, 2013; Baumgart and Baumgart, 2016). The mouse brains were harvested at E18.5, and 20 μm thick Cryostat brain sections were immune-stained with the anti-GFP antibody and fluorescence-conjugated secondary antibody. Images were collected using a confocal microscope. GFP-positive cells in the different cortical layers were analyzed using ImageJ. All animal experiments were approved by the Animal Ethics Committee of Peking University First Hospital.

### Statistical Analysis

All experimental data were processed using SPSS 25.0 for Mac and GraphPad Prism statistical software. The normal distribution data of the continuous variables were represented by the mean ± standard deviation (SD). A student’s *t*-test was used for comparison between two groups, and a one-way ANOVA was used for comparison between three groups and more than three groups. An LSD analysis was used for comparisons between the two groups. A *P* < 0.05 was considered statistically significant.

## Results

### Clinical Characteristics of a Patient With the Brain *RALA* c.G482A Somatic Variant

**FIGURE 1:**
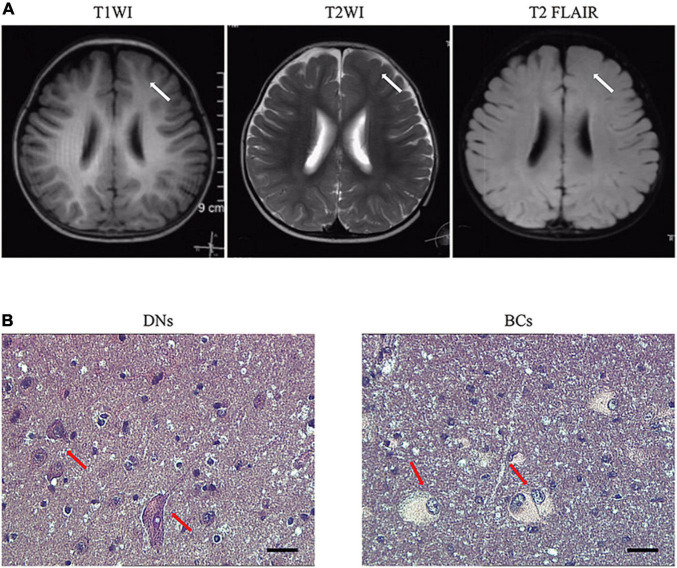
Representative MRI and pathology of patients with *RALA* c.G482A somatic variant. **(A)** MRI axial representations of the *RALA* somatic mutation patient. T1WI, T1-weighed image; T2WI, T2-weighed image; T2 FLAIR, T2 fluid-attenuated inversion recovery. The location of the lesion is indicated by a white arrow. **(B)** Histopathological results of the patient. Dysmorphic neurons and balloon cells are shown in the red arrow. HE, 10 × 40; DNs, dysmorphic neurons; BCs, balloon cells.

Male focal seizures began at 8 months of age, and these were characterized by squinting of the eyes to the left, clenching of the right hand, flexion, and stiffness of the right limb lasting for several minutes, and relieving by dozens to hundreds of attacks per day. The patient was treated with valproic acid, clonazepam, levetiracetam, and oxcarbazepine successively, which were poorly controlled and accompanied by severe developmental retardation. The video electroencephalogram (EEG) monitored multiple left frontopolar onset seizures during the waking hours. The cranial magnetic resonance imaging (MRI; Figure 1A) suggested dysplasia of the left frontal cortex. At the age of 1 year and 11 months, the child underwent resection of the left frontal epileptogenic focus, and the postoperative pathology suggested FCD Type IIb (Figure 1B). There was no recurrence of epilepsy at the 3-year postoperative follow-up, and there existed an Engel grade of I. Development was also significantly improved compared with the preoperative period.

### *RALA* Variation Site and the RalA Protein Structure

**FIGURE 2:**
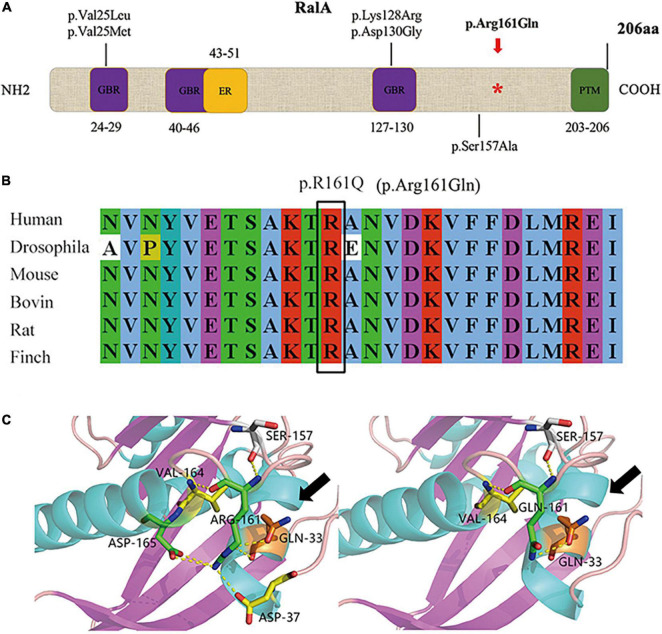
Domain diagram of human RalA and *RALA* variants. **(A)** Domains of RalA. GBR, GTP/GDP binding regions; ER, effector region; PTM, posttranslational modification. A red asterisk *: mutation site of *RALA* we identified in FCD type II tissue. The other variants associated with neurological diseases are also shown. **(B)** Homology comparison between species of *RALA* variant. **(C)** Protein structure before and after mutation of *RALA* c.G482A (p.Arg161Gln), as indicated by the black arrow.

We identified the first somatic variant of *RALA* (c.G482A, p.Arg161Gln) in the brain tissue of FCD II in our previous study, which was not retrieved as a minor allele frequency in the dbSNP, ExAC, and GnomAD databases and was predicted to be pathogenic by various mutation hazard analysis software such as SIFT and PolyPhen-2. The VAF was 5.50% in the lesions and was not detected in the perilesional brain tissues or peripheral blood. RalA is a small GTPase encoded by the *RALA* gene, a member of the Ral subfamily of the Ras superfamily of proto-oncogenes. The RalA structural domain primarily consists of three components that include three GTP/GDP binding domains, one effector binding domain, and a posttranslational modification site (containing the phosphorylation site of serine/threonine kinase) located at the COOH end (Figure 2A). Analysis of the RalA secondary structural domain showed that the p.Arg161Gln variant was located near the GTP/GDP binding domain. The homology alignment of multispecies sequences revealed that the variation loci were relatively conservative among species (Figure 2B). In addition, a predicted comparison of the tertiary protein structure before and after the mutation showed that the *RALA* p.Arg161Gln variant changed the arginine to glutamine at position 161 of the RalA protein, and this may lead to a reduction in the binding of hydrogen bonds and loosening of the protein structure. This results in the exposure and activation of the GTP/GDP binding domain and promotes the binding and hydrolysis of GTP while activating the corresponding downstream effector molecules (Figure 2C).

### *RALA* c.G482A Increased the RalA Expression

**FIGURE 3:**
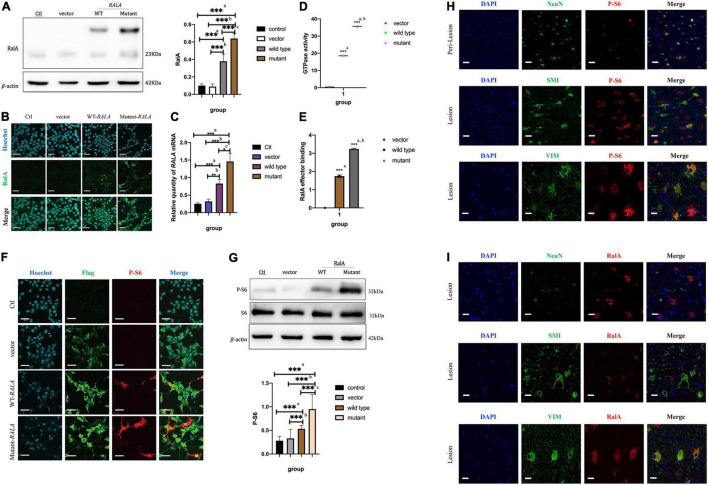
*RALA* c.G482A increases the expression of RalA and promotes mTOR pathway overactivation. **(A)** Expression of RalA protein between different groups after *RALA* c.G482A transfection. Ctl, control; WT, wild type of *RALA*; a, compared with Ctl; b, compared with vector; c, compared with WT. *n* = 6; ***P < 0.001. **(B)** Immunofluorescence of RalA protein in different groups after *RALA* mutant transfection. **(C)** Changes of *RALA* mRNA expression in different groups. *n* = 4; *P < 0.05; **P < 0.01; ***P < 0.001. **(D)** Detection of GTPase activity of RalA. a, compared with vector group; b, compared with wild type. *n* = 3; ***P < 0.001. **(E)** Downstream effector activation assay after *RALA* mutation. a, compared with vector group; b, compared with wild type. *n* = 3; ***P < 0.001. **(F,G)** Immunofluorescence staining and Western blotting showed the activation of the mTOR pathway. P-S6, phospho-S6 Ribosomal protein (Ser240/244), one of the typical markers of mTOR pathway activation. Bar = 100 μm; magnification: 10 × 60. *n* = 6; ***P < 0.001. **(H)** Activation of mTOR pathway in brain tissue. NeuN, marker of neurons; SMI-311, marker of dysmorphic neurons; Vimentin (V9), marker of balloon cells. **(I)** Expression of RalA protein in brain tissue. Bar = 50 μm; magnification: 10 × 40.

After transfection of the HEK293T cells *in vitro*, we found that transfection of the *RALA* c.G482A mutant significantly increased the expression of the RalA protein. Compared with the vector group (0.64 ± 0.06 vs. 0.09 ± 0.04, *P* = 0.000, *n* = 6) and the wild-type group (0.64 ± 0.06 vs. 0.38 ± 0.08, *P* = 0.000, *n* = 6), the RalA protein expression was significantly increased in the *RALA* mutant group (Figure 3A). In addition, the immunofluorescence staining also exhibited consistent results (Figure 3B). The mRNA expression was also significantly upregulated in the *RALA* c.G482A group compared with the vector group (1.46 ± 0.23 vs. 0.32 ± 0.06, *P* = 0.000, *n* = 4) and the wild-type group (1.46 ± 0.23 vs. 0.83 ± 0.12, *P* = 0.024, *n* = 4) (Figure 3C).

### *RALA* c.G482A Led to Increased RalA GTPase Activity and the Activation of Downstream Effectors

To illustrate the effect of this variant on RalA GTPase activity and downstream effectors, subsequent experiments were conducted. According to the characteristic tags carried by the transfected plasmids, Flag-tagged protein purification was first performed on the extracted total cell proteins using specific DYKDDDDK magnetic agar to obtain different groups of RalA proteins. Following that, the RalA GTPase activity was assayed using the Ral GTPase kit. The results demonstrated that the RalA GTPase activity was enhanced in the *RALA* c.G482A group compared with the vector group (35.68 ± 0.53 vs. 0.37 ± 0.20, *P* = 0.000, *n* = 3) and the wild-type group (35.68 ± 0.53 vs. 18.54 ± 0.39, *P* = 0.000, *n* = 3) (Figure 3D).

Then, RalA downstream effector binding assays were implemented based on the purified proteins harvested above. The results showed that the downstream effector binding was also significantly increased (Figure 3E) compared with the vector group (3.2384 ± 0.0436 vs. 0.0017 ± 0.0010, *P* = 0.000, *n* = 3) and the wild-type transfected group (3.3284 ± 0.0436 vs. 1.7440 ± 0.0719, *P* = 0.000, *n* = 3).

The above results indicated that *RALA* c.G482A brought about a significant increase in RalA GTPase activity as well as activation of downstream effectors. This implied that the *RALA* c.G482A mutation resulted in a gain of function.

### *RALA* c.G482A Caused Aberrant Activation of the Mammalian Target of Rapamycin Pathway

To verify whether the *RALA* mutation leads to mTORC1 overactivation, we examined the level of ribosomal S6 protein phosphorylation in HEK293T cells after transfection, a typical marker of mTOR pathway activation. We observed that cells expressing the *RALA* c.G482A mutant had significantly higher levels of S6 phosphorylation compared with wild-type or vector-transfected group cells (Figures 3F,G). These results suggest that the *RALA* c.G482A mutation leads to abnormal activation of the mTOR pathway.

Immunofluorescence staining was performed on the brain tissue sections of patients carrying the somatic variant of *RALA* c.G482A labeled with NeuN, SMI-311, and Vimentin (V9) for neurons, DNs, and BCs, respectively. The phosphorylation level of the S6 protein was significantly higher at the FCD lesion than in the perilesional tissues and colocated with DNs and BCs (Figure 3H), indicating the overactivation of the mTOR pathway in the patient’s brain tissue, which was consistent with the results of the *in vitro* cellular experiments. Then, we detected the expression and localization of the RalA protein in the lesioned brain tissue, the results proved that the expression of the RalA protein was significantly colocalized with BCs and DNs, and the RalA expression was more enriched in the BCs than the DNs (Figure 3I).

### *RALA* c.G482A Might Mediate Activation of Downstream Mammalian Target of Rapamycin Pathways via Downstream Effectors Phospholipase D1 or EXOC2

**FIGURE 4:**
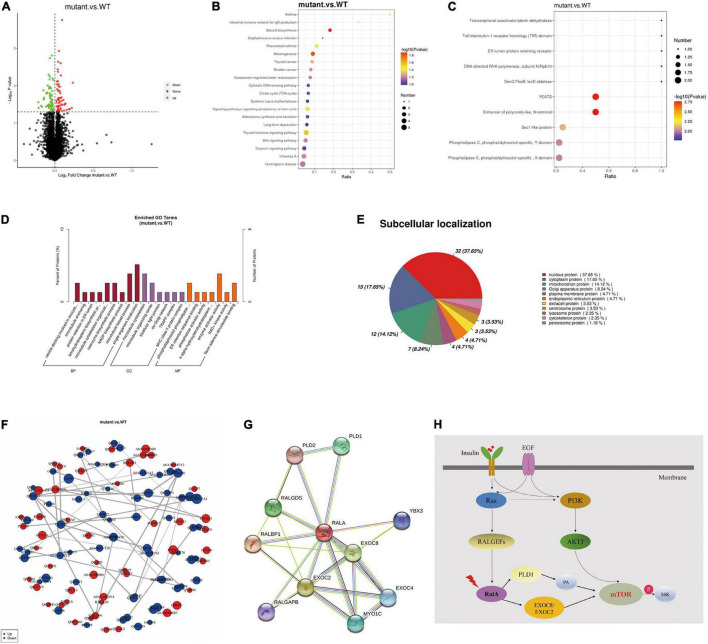
Tandem mass tag (TMT) quantitative proteomics assay. **(A)** Differential proteins volcano map. The horizontal axis represents the multiple of difference (Log2 value) of differential proteins, the vertical axis represents *P*-value (–log10 value), the black represents the protein with no significant difference, the red represents the upregulated protein, and the green represents the downregulated protein. **(B)** Kyoto Encyclopedia of Genes and Genomes (KEGG) enrichment bubble chart. The abscissa in the figure is the ratio of the number of differential proteins in the corresponding pathway to the total number of proteins identified in the pathway. The size of the point represents the number of differential proteins in the corresponding pathway. The larger the point, the more differential proteins in the pathway. **(C)** Interproscan software enrichment scatterplot. The abscissa is the ratio of the number of differential proteins in the corresponding domain to the total number of proteins identified in the domain. The size of the point represents the number of differential proteins in the corresponding domain. The larger the point, the more differential proteins in the domain. **(D)** Gene ontology enrichment analysis histogram. BP, GO_Class, biological process; CC, GO_Class, cellular component; MF, GO_Class, molecular function. *x*, the number of differential proteins associated with the gene ontology (GO); *n*, number of differential proteins annotated by GO. The diagram shows the enrichment of the three categories as a result, each show at most 20 (*P*-value ≤ 0.05), and the percentage of the ordinate represents the *x*/*n* in the table. **(E)** Subcellular localization analysis of differential proteins. **(F)** Differential protein-protein interaction analysis. Each node in the interaction network represents a protein, and the node size represents the number of proteins interacting with it. The larger the node, the more the proteins interacting with it. The color of the node indicates the expression level of this protein in the comparison pair. The red color represents the upregulated proteins, and the blue color represents the significantly downregulated proteins. **(G)** Top 10 proteins that interact with RalA, identified using the String DB protein interaction database (http://string-db.org/). **(H)** RALA somatic variant might activate mTOR pathway through downstream effectors PLD1 or EXOC8/EXOC2.

Furthermore, to inquire about the pathway through which RalA activates the mTOR pathway, we further conducted quantitative proteomic analysis on the transfected cells of *RALA* wild-type and *RALA* mutant. A total of 10 FC > 1.2-fold differentially expressed proteins (DEPs) were identified in the mutant and wild-type groups by proteomic mass spectrometry, with a total of 7,208 proteins detected in each sample. Gene ontology (GO) enrichment analysis and Kyoto Encyclopedia of Genes and Genomes (KEGG) enrichment results of DEPs are shown in Figures 4A–F, which are mainly involved in regulating cytoskeleton assembly, microtubule movement, phospholipase activity, and docking of exocytosis vesicles. Finally, we used the String DB protein interaction database to map the top 10 protein molecules that directly interact with RalA (Figure 4G and Table 1) and hypothesized that *RALA* c.G482A may be involved in the regulation of cell polarity and cell migration through the downstream pathway of phospholipase D1 (PLD1)-mediated activation of mTOR, or through EXOC8/EXOC2 (Figure 4H) mediated exocyst complex to recruit downstream kinases, which in turn leads to aberrant activation of the mTOR pathway (Zaman et al., 2021). Whether the *RALA* c.G482A variant mediates downstream mTOR pathway activation *via* PLD1/EXOC2 is currently only a hypothesis and requires further experimental validation.

**TABLE 1:** A list of the top 10 proteins interacting with RalA.

Predicted partners	Functional annotations
RalA-binding protein 1 (RalBP1)	As a downstream effector of RalA and RalB. As a GTPase-activating protein/GAP inactivated CDC42 and RAC1. As an effector of RalA controlling mitochondrial fission in mitosis. Regulating ligand-dependent EGF and insulin receptors-mediated endocytosis.
Exocyst complex component 2 (EXOC2)	Component of the exocyst complex involved in the docking of exocytic vesicles. Belongs to the SEC5 family.
Exocyst complex component 8 (EXOC8)	Component of the exocyst complex involved in the docking of exocytic vesicles. Belongs to the EXO84 family.
Ral guanine nucleotide dissociation stimulator (RALGDS)	Stimulating GTP binding and activation of the RalA and RalB GTPases. Interacts and acts as an effector molecule for R-Ras, H-Ras, K-Ras.
Phospholipase D1 (PLD1)	Implicated in signal transduction, membrane trafficking, and the regulation of mitosis.
Unconventional myosin-Ic (MYO1C)	Actin-based motor molecules with ATPase activity served in intracellular movements. Involved in glucose transporter recycling by regulating movement of intracellular GLUT4-containing vesicles to the plasma membrane. Acts as a mediator of adaptation of mechanoelectrical transduction.
Exocyst complex component 4 (EXOC4)	Component of the exocyst complex involved in the docking of exocytic vesicles with fusion sites on the plasma membrane.
Y-box-binding protein 3 (YBX3)	Binds to the GM-CSF promoter as a repressor. Binds to full-length mRNA and to short RNA sequences containing the consensus site 5′-UCCAUCA-3′ acted as translation repression.
Ral GTPase-activating protein subunit beta (RALGAPB)	Non-catalytic subunit of the heterodimeric RalGAP1 and RalGAP2 complexes. GTPase activators for the Ras-like small GTPases RALA and RALB.
Phospholipase D2 (PLD2)	Involved in signal-induced cytoskeletal regulation and/or endocytosis.

### *In utero* Electroporation of the *RALA* c.G482A Somatic Mutation-Induced Migration Disorders of Mice Cortex Neurons

**FIGURE 5:**
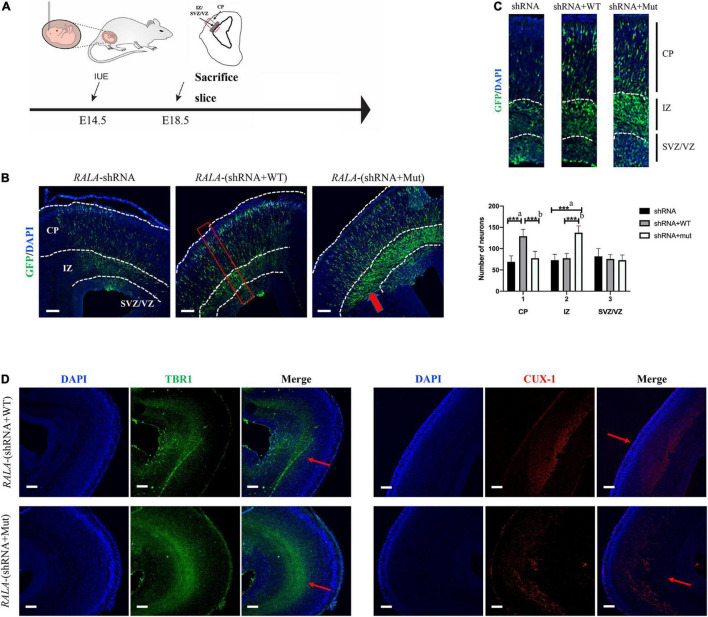
Simulation of cortical dysplasia in mouse models *via in utero* electroporation. **(A)** Schematic diagram of animal experiment. **(B)** *RALA* c.G482A causes disorders of cortical neuron migration, as indicated by the thick red arrow. **(C)** Count and statistics of neurons in different areas of mouse cortex. Local amplification figure from the red square area. **(D)** Immunofluorescence labeled layer II/III/IV and layer V/VI neurons, thin red arrows. IUE, *in utero* electroporation; E14.5, embryo 14.5 days; VZ, ventricular zone; SVZ, subventricular zone; IZ, intermediate zone; CP, cortical plate; EEG, Electroencephalogram; shRNA, short hairpin RNA; TBR1, T-box brain transcription factor 1; CUX-1, Cut Like Homeobox 1. a, compared with *RALA*-shRNA group; b, compared with *RALA*-(shRNA + WT) group. *n* = 6, ***P < 0.001. Bar = 10 μm; magnification: 10 × 10.

We electrotransfected wild-type or *RALA* c.G482A IRES-GFP plasmids into E14.5 mice embryos (Figure 5A) and sacrificed the mice at E18.5 for brain slices to measure the radial migration of GFP-positive cells in the cortex and the distribution of neurons in different cortical areas. The immunofluorescence results showed that neurons in the *RALA* c.G482A group migrated less from the paraventricular to the cortical plate (CP) and were more concentrated in the intermediate zone (IZ) compared with the wild-type group (Figure 5B). Neurons were counted in different parts of the mouse cerebral cortex (Fata et al., 2014), and the results displayed that the number of neurons migrating to the CP was significantly decreased in the *RALA* c.G482A group mice compared with the wild-type group (77.33 ± 16.21 vs. 129.00 ± 16.46, *P* = 0.000, *n* = 6), while the number of neurons in the IZ region was significantly increased (137.33 ± 15.96 vs. 77.17 ± 11.41, *P* = 0.000, *n* = 6). The number of neurons in the subventricular area (SVZ) in the two groups was 72.83 ± 12.14 vs. 75.50 ± 10.33 (*P* = 0.747, *n* = 6), and the difference was not statistically significant (Figure 5C). Mice expressing *RALA* c.G482A had reduced numbers of GFP-positive cells in the CP areas and increased numbers of GFP-positive cells in the ventricular and paraventricular areas, suggesting that the expression of *RALA* c.G482A can lead to neuronal migration disorders.

To further confirm the effect of *RALA* variation on neuronal migration, we labeled the superficial layer II–IV neurons (CUX-1) and the deep layer V/VI neurons (TBR1) separately, and the staining results revealed that more cortical neurons in the mutant group resided in the layer V/VI cortex and fewer neurons migrated to the layer II–IV compared with the wild-type mice (Figure 5D).

## Discussion

*RALA* encodes a small GTPase of the Ras superfamily, RalA, that is widely expressed in various tissues throughout the body and is involved in a series of biological functions, such as gene expression, cell migration, cell proliferation, and membrane transport by interacting with different downstream effectors (Yan and Theodorescu, 2018). In the CNS, RalA is engaged in neuronal development, plasticity, polarization, migration, synaptic vesicle fusion, NMDA, AMPA, and dopamine receptor regulation (Teodoro et al., 2013; Zheng et al., 2016).

Our group first detected the *RALA* somatic variant in FCD II brain tissue using whole-exome sequencing (WES), and we verified this using amplicon sequencing with a VAF of 5.50%. In this study, we discovered that the *in vitro* transfection of the *RALA* c.G482A variant resulted in increased RalA protein expression and RalA GTPase activity and the activation of its downstream effectors. In addition, we observed an obvious increase in S6 phosphorylation and overactivation of the mTOR pathway after transfection. Immunofluorescence staining of the brain tissue specimens from this patient also confirmed the overactivation of the mTOR pathway in brain tissue, and that the *RALA* c.G482A variant was more enriched in the BCs and DNs. Then, by constructing an *in vivo* electrotransfected mice model, we found that the *RALA* c.G482A variant apparently affected cortical neuronal migration, and this resulted in more cortical neurons residing in the paraventricular region and the layer V/VI cortex and less migration in the superficial II-IV cortex. These results suggest that in FCD type II brain tissue lesions, the somatic variant of the *RALA* gene is primarily distributed in BCs and DNs, and this variant leads to the functional gain of RalA, activating its downstream effector molecules, further activating the PI3K-AKT-mTOR pathway, thus affecting neuronal migration, and ultimately leading to the development of FCD.

Somatic variants of *RALA* have been previously reported in cancers, and a total of 492 *RALA* somatic variants have been found in various tissues and organs, suggesting that pathogenic *RALA* somatic variants are associated with a wide range of disease phenotypes. Among them, the *RALA* c.G482A somatic variant we identified has been reported to be associated with adult T-cell lymphoma leukemia (Kataoka, 2015) and malignant melanoma (Hayward et al., 2017; Wilmott et al., 2019), and no studies have been performed to associate them with neurological diseases. Moreover, Hiatt et al. (2018) reported cases of *de novo RALA* germline variants causing Hiatt-Neu-Cooper neurodevelopmental syndromes (HINCONS) with the variation that included six missense variants of c.G73A, c.G73T, c.A383G, c.A389G, c.T469G, and c.C526T and one chromosomal microdeletion of c.472_474delGCT. HINCONS is an autosomal dominant neurodevelopmental disorder characterized by generalized developmental delay, intellectual disability, language deficits, and facial dysmorphism. It may also be accompanied by epilepsy, autism, and structural brain abnormalities, such as corpus callosum dysplasia and polymicrogyria. The phenotypic heterogeneity between germline variants and somatic variants of the same gene has also been appeared in other variants associated with neurological disorders. Bonduelle et al. (2021) investigated 20 cases of mild malformations of cortical development with oligodendroglial hyperplasia in epilepsy (MOGHE) children using brain tissue sequencing. *SLC35A2* somatic mutations were detected in 9/20 (45%) patient brains, while the germline variants of the *SLC35A2* gene led to developmental and epileptic encephalopathies. Their findings highlight the importance of brain somatic mutations in the etiology of focal epilepsy associated with MCD.

Currently, the genes located in the mTOR pathway identified in the FCD II brain tissues are classified into three types: (Mariasavina et al., 2020) gain-of-function variation in the mTOR upstream regulatory genes, such as *PIK3CA*, *AKT3*, and *RHEB*; (Ingmar et al., 2011) loss-of-function variation of the mTOR pathway suppressor genes, such as *DEPDC5*, *NPRL2*, *NPRL3*, and *PTEN*; and (Blumcke et al., 2017) somatic gain-of-function mutations in the *MTOR* gene. Several of these genes also have somatic second-hit mutations, including *AKT3*, *DEPDC5*, *MTOR*, *PIK3CA*, *RHEB*, *TSC1*, and *TSC2*. Jansen et al. (2015) identified *AKT3* and *PIK3CA* somatic mutations in the brain tissue of FCD II patients using targeted sequencing of 10 genes of the mTOR pathway. Mirzaa et al. (2016) performed WES of lesioned brain tissues and peripheral blood samples to find *MTOR* somatic mutations, and these somatic pathogenic variants were enriched in the DNs and BCs, suggesting that DNs and BCs may be the few abnormally differentiated somatic cells that carry pathogenic variants. Our group (Zhang et al., 2020) previously screened seven potential FCD II-related somatic variants using WES and amplicon sequencing in 6/17 (35%) brain tissue specimens from FCD II patients. Except for *MTOR* and *TSC2*, which are the reported genes, the remaining five genes, *IRS1*, *ZNF337*, *HTR6*, *RALA*, and *RAB6B*, were not yet associated with FCD. Nevertheless, no clear pathogenic somatic mutations have been detected in the brain tissue of more than half of the patients. Therefore, it remains to be determined whether other genetic variants in the upstream pathway related to mTOR and abnormal regulation of other signaling pathways are also implicated in the pathogenesis of FCD. Our findings suggest that there are other genetic variants in FCD II brain tissue. However, our findings show that *RALA* c.G482A can also activate the mTOR pathway, and we hypothesized that the *RALA* variant can lead to the aberrant activation of the mTOR pathway through PLD1-mediated downstream signaling pathways. Several previous studies have also reported that activation of PLD1, a downstream effector of RalA, can lead to an increased intracellular phosphatidic acid concentration that in turn activates the mTOR pathway (Xu et al., 2011; Bernfeld et al., 2018). Therefore, mTOR pathway activation may also be a common pathway for FCD II pathogenesis.

However, there are still some limitations in this study: (Mariasavina et al., 2020) all of the FCD II-related somatic variant genes discovered and reported so far are in the mTOR pathway and its regulatory pathways, including the *RALA* gene that we newly identified in this study, and no variant of genes in other pathways outside this pathway has been found (Ingmar et al., 2011). Due to the limitation of the sequencing depth and cost of WES, the somatic variants were only detected in a small number of FCD II brain tissue samples, among which the *RALA* somatic variant was detected in the brain tissue of only one FCD IIb patient, and more than half of the samples were still negative for genetic testing, pending further sequencing (Blumcke et al., 2017). Although our study found that the *RALA* c.G482A variant activated the mTOR pathway and caused cortical neuronal migration disorders, considering that children carrying this variant have epilepsy, further studies are required to better correlate genotype with phenotype as to how *RALA* c.G482A causes FCD and subsequently affects the electrophysiological properties of neurons, ultimately leading to epilepsy.

In summary, we investigated the role of *RALA* gene dysfunction in the formation of FCD II using functional studies of the newly identified brain *RALA* somatic variant at the cellular and animal levels in terms of their effects on the PI3K-AKT-mTOR signaling pathway and on cortical neuronal migration, thus confirming that *RALA* is a new pathogenic gene related to FCD II. The results of the study help to expand the understanding of the pathogenesis of FCD II, enrich the gene spectrum of somatic variants associated with MCD, and provide a basis for clarifying the mechanism of FCD II to explore new therapeutic targets. However, the brain *RALA* somatic variant was found in only one patient with FCD II, and this result needs to be confirmed in more patient samples.

## Data Availability Statement

The original contributions presented in this study are included in the article/supplementary material, further inquiries can be directed to the corresponding authors.

## Ethics Statement

The studies involving human participants were reviewed and approved by the Ethics Committee of Peking University First Hospital. Written informed consent to participate in this study was provided by the participants’ legal guardian/next of kin. The animal study was reviewed and approved by the Animal Ethics Committee of Peking University First Hospital. Written informed consent was obtained from the individual(s), and minor(s)’ legal guardian/next of kin, for the publication of any potentially identifiable images or data included in this article.

## Author Contributions

HX was the executor of this study, who completed experiment procedures, and manuscript writing. KG, QL, TW, ZZ, and LC participated in clinical data collection and result analysis. YW and YJ were the corresponding authors in charge of the study and directed the experimental design, data analysis, manuscript writing, and revision. All authors agreed to be accountable for the content of this work.

## Conflict of Interest

The authors declare that the research was conducted in the absence of any commercial or financial relationships that could be construed as a potential conflict of interest.

## Publisher’s Note

All claims expressed in this article are solely those of the authors and do not necessarily represent those of their affiliated organizations, or those of the publisher, the editors and the reviewers. Any product that may be evaluated in this article, or claim that may be made by its manufacturer, is not guaranteed or endorsed by the publisher.
